# Phenotype and Viability of MLO-Y4 Cells Is Maintained by TGFβ_3_ in a Serum-Dependent Manner within a 3D-Co-Culture with MG-63 Cells

**DOI:** 10.3390/ijms19071932

**Published:** 2018-06-30

**Authors:** Katharina Jähn, Deborah J. Mason, Jim R. Ralphs, Bronwen A.J. Evans, Charles W. Archer, R. Geoff Richards, Martin J. Stoddart

**Affiliations:** 1 Department of Osteology and Biomechanics, University Medical Center Hamburg-Eppendorf, 22529 Hamburg, Germany; 2 School of Biosciences, Cardiff University, CF10 3AX Cardiff, UK; masondj@cardiff.ac.uk (D.J.M.); ralphs@cardiff.ac.uk (J.R.R.); geoff.richards@aofoundation.org (R.G.R.); 3 School of Medicine, Cardiff University, CF14 4XN Cardiff, UK; evansba@cardiff.ac.uk; 4 College of Medicine, Swansea University, SA2 8PP Swansea, UK; c.archer@swansea.ac.uk; 5 AO Research Institute Davos, AO Foundation, 7270 Davos, Switzerland; martin.stoddart@aofoundation.org

**Keywords:** bone biology, osteoblast, osteocyte, three-dimensional (3D) co-culture, transforming growth factor-beta 3 (TGFβ_3_)

## Abstract

The osteocyte network inside the bone matrix is of functional importance and osteocyte cell death is a characteristic feature of pathological bone diseases. Osteocytes have emerged as key regulators of bone tissue maintenance, yet maintaining their phenotype during in vitro culture remains challenging. A 3D co-culture system for osteocytes with osteoblasts was recently presented, enabling the determination of more physiological effects of growth factors on cells in vitro. MLO-Y4 cells were embedded within a type I collagen gel and cultured in the presence of surface MG-63 cells. Co-culture was performed in the presence or absence of TGFβ_3_. Gene expression by quantitative PCR, protein expression by fluorescent immunohistochemistry and cell viability tests were performed. The 3D co-culture induced cell differentiation of MG-63 cells seen by increased type I collagen and osteocalcin mRNA expression. TGFβ_3_ maintained osteocyte differentiation of MLO-Y4 cells during co-culture as determined by stable E11 and osteocalcin mRNA expression till day 4. Interestingly, most of the effects of TGFβ_3_ on co-cultured cells were serum-dependent. Also, TGFβ_3_ reduced cell death of 3D co-cultured MLO-Y4 cells in a serum-dependent manner. This study shows that 3D co-culture upregulates differentiation of MG-63 cells to a more mature osteoblast-like phenotype; while the addition of TGFβ_3_ maintained the characteristic MLO-Y4 osteocyte-like phenotype and viability in a serum-dependent manner.

## 1. Introduction

Members of the Transforming growth factor beta (TGFβ) super family mediate key events in skeletal development and postnatal bone homeostasis, acting on osteoblasts, osteoclasts, as well as the crosstalk between both cell types [1]. TGFβs are synthesized as large precursor molecules, containing both the active peptide and a non-covalently bound latency-associated protein. In this latent form, TGFβs are stored in vast amounts in the extracellular matrix (ECM) of bone [2,3,4]. Their release and activation from the ECM is dependent upon the bone resorptive activity of osteoclasts at active sites of bone remodelling [5]. Once released, the effects of TGFβs on osteoblasts are multifactorial and dependent on their stage of differentiation [6]. It has long been known that TGFβs act on osteoblast progenitors as a chemoattractant, initiating their migration during a bone remodelling cycle, or in the case of fracture repair towards sites of new bone formation [7]. TGFβ induces osteoblasts to proliferate, differentiate and produce extracellular matrix [8,9]. However, the effects of TGFβ on cells of the osteoblast lineage are complex and even though TGFβ increases osteoid production, it inhibits mineralization of the produced matrix [10]. Consequently, type I collagen expression is stimulated by TGFβ, while osteocalcin expression is inhibited by TGFβ [11].

During bone formation, some osteoblasts are encased and terminally differentiate further to dendritic osteocytes. These lie within a three-dimensional (3D) fluid-filled lacuno-canalicular system that spans through the entire bone matrix and predisposes osteocytes as the mechanosensoric cells in bone tissue [12]. Recently, osteocytes have come to the spotlight of research projects and their established multifunctionality ranges from regulation of phosphate homeostasis, to directing bone turnover and even influencing distant tissue functions (for review see: [13,14]). With osteocytes being buried alive within bone, osteocyte viability is of essential importance to the maintenance of bone homeostasis. They are thought to be one of the longest living cells in our body. We have previously shown that TGFβ_3_ is able to maintain osteocyte viability in an ex vivo culture of human cancellous bone explants [15,16]. Within cancellous bone explants, it was demonstrated that the positive cell survival effect by daily dynamic compressive load was enhanced by the addition of TGFβ_3_. Apart from the clear advantage of a bone organ culture, namely the naturally occurring cell and matrix interactions, the presence of a highly calcified extracellular matrix complicates investigations of cellular reactions, the culture requires an advanced bioreactor setup and the maintenance of the cell culture conditions is not trivial [17].

Discoveries on the in vitro effects of TGFβs on cultured osteoblast-like cells have been performed mainly using monolayer cultures. Yet, (i) bone is a 3D tissue which is characterized by extensive cell-to-cell and cell-to-matrix connectivity, that is, the dendritic osteocyte network embedded in the bone matrix interacting with surface osteoblasts and (ii) it is not yet clear if these could alter the effect, effect size or timing of cellular responses to growth factors. The effects of growth factors in vivo will also be defined by such cellular interactions. Vazquez et al. [18] described a 3D co-culture method for surface osteoblast-like cells and collagen-matrix embedded osteocyte-like cells. The authors presented a system consisting of osteoblasts layered on top of a type I collagen gel, which contains 3D-embedded, interconnected osteocytes. Within the model system both cell types were shown to express appropriate phenotypic markers for up to 7 days and osteocytes were found to respond to mechanical stimulation. This allows the effects of cytokines on osteoblasts and on osteocytes to be tested in the presence of a 3D osteoblast-osteocyte-collagen matrix connectivity.

In this study, we aim to investigate the effects of TGFβ_3_ on both osteoblasts (MG-63 cell line) and osteocytes (MLO-Y4 cell line) within a 3D co-culture. Moreover, a defined serum-free (SF) culture medium was created to facilitate the serum-independent investigation of the cellular effects of TGFβ_3_ on cell phenotype and viability.

## 2. Results

### 2.1. TGFβ_3_ Maintained Runx2 Expression in Co-Cultured MG-63 Osteoblast-Like Cells and Did not Affect the Increase in Osteocalcin Expression

**Figure 1:**
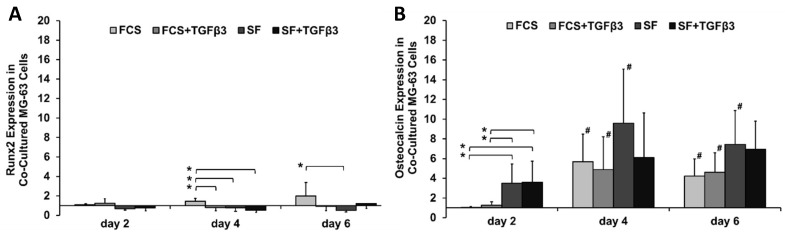
Fold-change in relative gene expression for (**A**) Runx2 (CT: 18–22) and (**B**) osteocalcin (CT: 18–24) in co-cultured MG-63 osteoblast-like cells. Gene expression levels were normalized to GAPDH and were made relative to cultures in αMEM + FCS on day 2. The dCT data was used for statistical analysis: normally distributed, one-way ANOVA with Tukey (*n* = 3–4 replicates/experiment, 3 experiments). Statistical significance was determined as *p* < 0.05; *: compared to different media on that day; #: compared to same medium group on day 2. Bar charts show the average fold-change as well as the standard error of the mean.

To investigate the effect of TGFβ_3_ on MG-63 osteoblast-like cells within a 3D osteoblast-osteocyte co-culture model, the expression of Runx2 and osteocalcin mRNA levels were evaluated. Runx2 is the major osteoblast transcription factor expressed early in osteoblast differentiation, while osteocalcin is expressed in large amounts by late osteoblasts, as well as early osteocytes and coincides with mineralization of the extracellular matrix [19]. All groups demonstrated similar Runx2 expression except for FCS alone, where *Runx2* levels increased steadily over time, leading to a significantly lower Runx2 expression in the MG-63 cell line with TGFβ_3_ + FCS compared to FCS alone at day 4 (Figure 1A).

Osteocalcin expression generally increased over time in co-cultured MG-63 osteoblast-like cells, with SF cultures exhibiting the largest increase. TGFβ_3_ did not influence osteocalcin mRNA expression (Figure 1B). This result suggests overall induction of osteoblast differentiation during culture, with the highest level of osteocalcin expression seen on day 4 using SF media alone. On day 4, only the addition of TGFβ to SF medium led to a non-significant increase in osteocalcin expression compared to significant increases in all other groups.

Serum-dependency: TGFβ_3_ prevented the increase in Runx2 expression in MG-63 cells during culture. This effect was only seen in the presence of FCS. While for osteocalcin expression, the effect of TGFβ_3_ was mostly serum-independent.

### 2.2. TGFβ_3_ Maintained Osteocalcin Expression in 3D Co-Cultured MLO-Y4 Cells Serum-Independently and E11 Expression in a Serum-Dependent Manner

**Figure 2:**
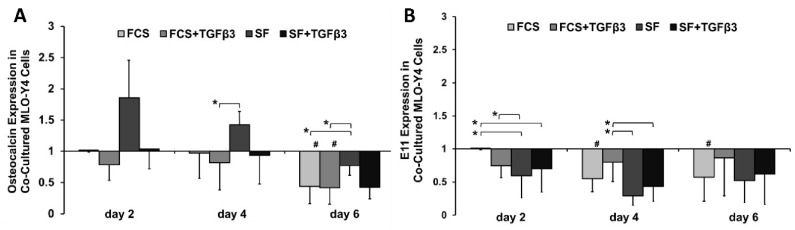
Fold-change in relative gene expression for (**A**) osteocalcin (CT: 18–23) and (**B**) E11 (CT: 19–21) in 3D co-cultured MLO-Y4 osteocyte-like cells. Gene expression levels were normalized to 18SrRNA and made relative to αMEM + FCS on day 2. The dCT data was used for statistical analysis: normally distributed, one-way ANOVA with Tukey (*n* = 3–4 replicates/experiment, 3 experiments). Statistical significance was determined as *p* < 0.05; *: compared to different media on that day; #: compared to same medium group on day 2. Bar charts show the average fold-change as well as the standard error of the mean.

To investigate the MLO-Y4 osteocyte phenotype during 3D co-culture with MG-63 osteoblast-like cells, we investigated mRNA expression of the late osteoblast marker osteocalcin and the early osteocyte marker E11. MLO-Y4s osteocalcin expression was maintained until day 4 by TGFβ_3_ in an FCS-independent manner, while SF-medium alone led to higher osteocalcin expression levels on day 2 and day 4 (Figure 2A). By day 6, osteocalcin expression was reduced in FCS-cultured MLO-Y4 osteocyte-like cells compared to SF alone. This indicates that osteocalcin expression is reduced in the presence of serum or TGFβ.

The addition of TGFβ_3_ to serum-containing medium maintained E11 expression at a constant level until day 6. In all other groups, E11 mRNA expression significantly decreased during 3D co-culture in FCS-cultured MLO-Y4 cells (Figure 2B).

Serum-dependency: In the absence of serum TGFβ_3_ prevented the increase in osteocalcin expression in MLO-Y4 osteocyte-like cells. While TGFβ_3_’s effect on maintaining E11 expression was only seen in the presence of serum.

### 2.3. TGFβ_3_ Regulated Type I Collagen Expression in Co-Cultured MG-63 Osteoblast-Like Cells

**Figure 3:**
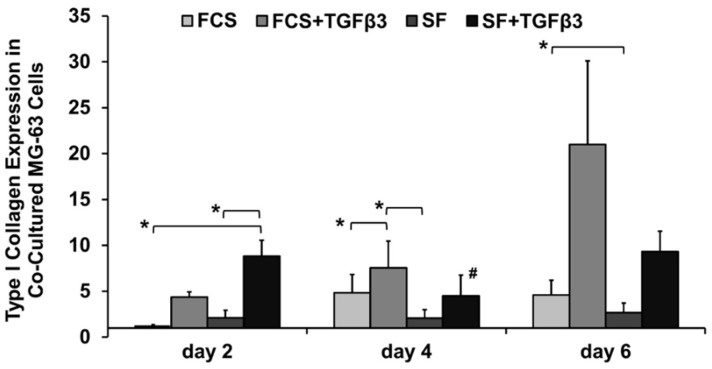
Fold-change in relative gene expression of type I collagen (CT: 21–31) in co-cultured MG-63 osteoblast-like cells. Gene expression levels were normalized to GAPDH and were made relative to αMEM + FCS on day 2. The dCT data was used for statistical analysis: normally distributed, one-way ANOVA with Tukey (*n* = 3–4 replicates/experiment, 3 experiments). Statistical significance was determined as *p* < 0.05; *: compared to different media on that day; #: compared to same medium group on day 2. Bar charts show the average fold-change as well as the standard error of the mean.

Type I collagen mRNA expression was solely evaluated in co-cultured MG-63 osteoblast-like cells, here TGFβ_3_ significantly affected collagen synthesis (Figure 3). Type I collagen was expressed at highest levels in the presence of TGFβ_3_. Yet, for serum-containing culture medium significance with TGFβ_3_ was reached on day 4, while SF culture showed significance upon TGFβ_3_ addition on day 2 (*p* = 0.045) (Figure 3).

Serum-dependency: TGFβ_3_ lead to higher type I collagen mRNA expression by MG-63 osteoblast-like cells in a serum-independent manner.

**Figure 4:**
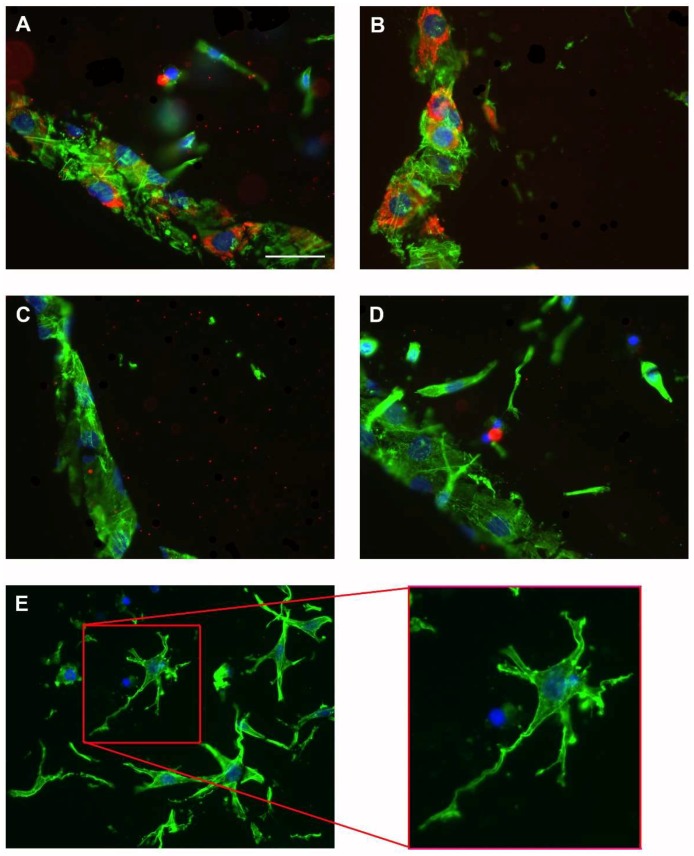
Fluorescent micrographs represent immuno-labelling of β-actin (Alexa 488, green) and the C-terminal propeptide of type I collagen (Alexa 594, red) in combination with DAPI nuclear staining (blue) (*n* = 4 replicates/experiment, 3 experiments). Shown are the micrographs for labelled surface MG-63 osteoblast-like cells on culture day 4 (**A**–**D**). (**A**) αMEM + FCS, (**B**) αMEM SF, (**C**) αMEM + FCS + TGFβ_3_, and (**D**) αMEM SF + TGFβ_3_. ProCI labelling in TGFβ_3_-containing medium was not yet present on day 4. MLO-Y4 osteocyte-like cells in co-culture demonstrated dendritic phenotype, these embedded cells inside the gel were negative for ProCI staining (**E**). Insert was magnified 2×. Scale bar represents 20 μm.

Immunocytochemical labelling of ProCI was used as qualitative analysis of type I collagen protein expression by co-cultured MG-63 osteoblast-like cells. Labelling revealed a difference in the onset of newly formed collagenous matrix deposition by the co-cultured MG-63s depending upon TGFβ_3_ presence. However, even though type I collagen mRNA expression was increased by TGFβ_3_ addition; on a protein level type I collagen synthesis was delayed if TGFβ_3_ was added to FCS or SF medium. ProCI was detected on culture day 2 on surface MG-63 osteoblast-like cells cultured in FCS or SF medium but only detected in TGFβ_3_ containing cultures at day 6 (Representative images at culture day 4 are shown in Figure 4). Co-cultured osteocytes were not positive for ProCI (Figure 4E).

Serum-dependency: The effect of TGFβ_3_ on type I collagen protein expression was serum-independent.

### 2.4. TGFβ_3_ Reduced Cell Death of MLO-Y4 Osteocyte-Like Cells in a Serum-Dependent Manner

E11 is an early osteocyte marker gene, associated with the development of the dendrites formed by osteocytes [20]. The connectivity of osteocytes within bone is crucial for osteocyte viability [21,22]. As TGFβ_3_ was found to maintain E11 gene expression of MLO-Y4 osteocyte-like cells during 3D co-culture, the influence of TGFβ_3_ on cell survival was investigated by LDH and TUNEL assays.

**Figure 5:**
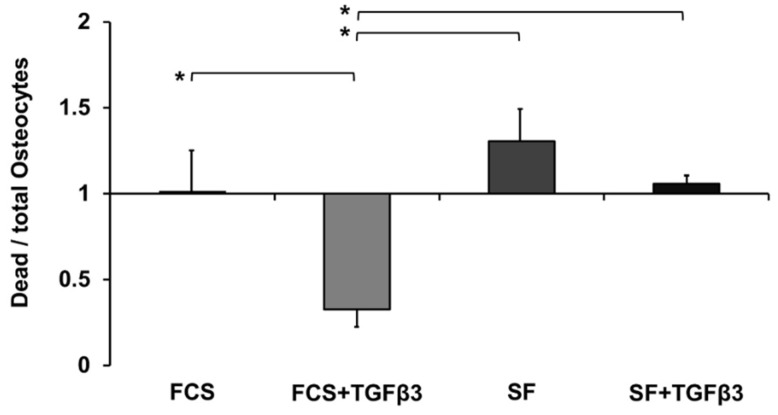
Graph to illustrate the effect of TGFβ_3_ on the viability of 3D co-cultured MLO-Y4 osteocyte-like cells. Data—collected on culture day 4—was normally distributed (*n* = 3 replicates/experiment, 3 experiments): one-way ANOVA and Tukey were used as statistical analysis. Statistical significance was defined as * *p* < 0.05. Bar chart shows the average osteocyte death and standard error of the mean.

A qualitative LDH activity assay showed the presence of viable MG-63 cells and MLO-Y4 cells under all culture conditions (data not shown). Quantitative TUNEL assay was performed to evaluate the effect of TGFβ_3_ on MLO-Y4 cell death within the 3D osteoblast-osteocyte co-culture system. TGFβ_3_ significantly increased MLO-Y4 osteocyte-like cell survival (Figure 5). Co-culture of MLO-Y4 osteocyte-like cells in medium containing both FCS and TGFβ_3_ showed the overall lowest cell death on day 4 of culture. The addition of TGFβ_3_ to SF medium did not increase MLO-Y4 cell viability.

Serum dependency: TGFβ_3_ reduced cell death of MLO-Y4 osteocyte-like cells at a later stage of co-culture only in the presence of serum.

## 3. Discussion

Within this paper we present that TGFβ_3_ affects differentiation and viability of MG-63 osteoblast-like cells and MLO-Y4 osteocyte-like cells within a 3D co-culture model. The co-culture facilitates the characteristic expression of type I collagen and osteocalcin mRNA by MG-63 osteoblast-like cells cultured as surface cells on top of a type I collagen gel, indicating that these cells undergo differentiation to a more mature osteoblast state during co-culture. At the same time, TGFβ_3_ maintained E11 expression of the embedded MLO-Y4 osteocyte-like cells. Most effects seen by TGFβ_3_ in co-culture were found to be serum-dependent, as was the prevention of apoptosis by TGFβ_3_ in co-cultured MLO-Y4 osteocyte-like cells.

Previous work has shown that the stimulatory effect of TGFβ_3_ on type I collagen and DNA synthesis, demonstrated previously in foetal rat osteoblast-enriched cultures, is more potent than using TGFβ_1_ [23]. The effect on type I collagen synthesis by TGFβs might be due to an increase in type I collagen mRNA stability, mRNA expression, or enhanced type I collagen synthesis [8,24]. Within this co-culture system we also demonstrated a significant up-regulation of type I collagen mRNA in the presence of TGFβ_3_. However, type I collagen protein synthesis appeared to be delayed by TGFβ_3_ and was only detectable at day 6. Thus, TGFβ_3_ increased type I collagen mRNA expression but must also activate post-transcriptional or post-translational processes to reduce pro-collagen synthesis or enhance pro-collagen degradation.

Within the 3D osteoblast-osteocyte co-culture, FCS-containing medium increased Runx2 mRNA expression in MG-63 osteoblast-like cells but this was prevented by the addition of TGFβ_3_. The results of several studies suggest that TGFβ induces initial pre-osteoblast proliferation and differentiation, while inhibiting terminal differentiation into a more mature osteoblast phenotype [1,6,24]. The down-regulation of Runx2 mRNA by TGFβ in mice calvaria-derived osteoblasts in the presence of serum was demonstrated previously [11]. Within our 3D co-culture study, we detected a down-regulation of Runx2 mRNA also exclusively in the presence of serum. The relative expression level of Runx2 caused by culture in αMEM + FCS + TGFβ_3_, or αMEM SF ± TGFβ_3_ might be seen as a transcriptional basal level within this co-culture system.

In contrast to previous mono-culture studies in 2D reporting the down-regulation of osteocalcin by TGFβ addition [11], within our 3D co-culture study TGFβ_3_ did not decrease osteocalcin mRNA in co-cultured MG-63 osteoblast-like cells. An increase in osteocalcin mRNA expression was detected over time in 3D co-cultured MG-63 osteoblast-like cells. The relative osteocalcin expression in the osteoblast-like cells was highest in serum free medium without the addition of the growth factor. On the contrary, embedded MLO-Y4 osteocyte-like cells significantly down-regulated osteocalcin mRNA expression in serum containing medium with or without TGFβ_3_.

E11 expression is a known marker for early osteocytes and its expression is significantly associated with dendrite formation and osteocyte responsiveness to fluid flow shear stress [20]. The MLO-Y4 osteocyte-like cell line as used in this study is characterized by a high expression level of E11 [25]. In this 3D co-culture investigation, we found that TGFβ_3_ maintained the expression of E11 mRNA in a serum-dependent manner, suggesting that TGFβ_3_ maintains the differentiation level of MLO-Y4 cells. E11 is an early osteocyte marker gene, associated with the development of the dendrites formed by osteocytes [20]. The connectivity of osteocytes via their dendritic network in bone is crucial for osteocyte viability [21,22].

The addition of TGFβ_3_ to αMEM + FCS significantly improved MLO-Y4 osteocyte survival. This effect was not visible in SF medium with TGFβ_3_. Suggesting that the combination of TGFβ_3_ with FCS is necessary to allow for superior cell viability during culture of the 3D construct. Karsdal et al. reported that TGFβ_1_ is able to rescue matrix metalloprotease-induced apoptosis of MC3T3-E1 cells [26]. MT1-MMP activation of latent TGFβ_1_ seems to be responsible for the maintenance of osteoblast viability during differentiation into osteocytes [27,28]. The influence of TGFβ_3_ on osteocyte survival was also demonstrated in an ex vivo culture system for human cancellous bone [13]. In addition, TGFβ has been shown to support osteocyte functionality [29]. To our knowledge, these previously reported cell culture experiments were performed in the presence of serum within the culture medium. Therefore, our results on the viability of 3D co-cultured MLO-Y4 osteocyte-like cells in αMEM + FCS + TGFβ_3_ confirms these previous studies.

Limitations: In addition to the growth factor and serum-dependent effects described, we have to acknowledge and mention that the serum-free medium used could also influence cellular responses. While we could only compare to FCS, which by itself is a mixture of growth factors and hormones, the insulin-transferrin-selenium-lipid supplementation used as serum-free medium contains potent molecules affecting osteoblast-lineage cell behaviour. However, this supplementation is widely established to be used in serum-free media and is needed to allow for cell viability.

Furthermore, we acknowledge the use of cell lines from two different donor species in our study. While it was our intention to be conform to the previously established protocol [18] using these, future studies should use cell lines derived from the same species and verify the results using primary derived cells.

Here we present for the first time the effects of TGFβ_3_ on 3D co-cultured MG-63 osteoblast-like cells and MLO-Y4 osteocyte-like cells. We were able to show that MG-63 cell differentiation was increased during 3D co-culture into a more mature osteoblast-like phenotype, and TGFβ_3_ maintained the characteristic MLO-Y4 osteocytic differentiation, while increasing MLO-Y4 cell survival in the 3D co-culture. The later effect confirmed our previous results of TGFβ_3_ on osteocyte viability in 3D cancellous bone explants cultured ex vivo in a bioreactor. The effects presented here were mostly found to be serum-dependent. Yet, further studies would be required to investigate the serum-dependency and prolong culture conditions beyond six days. The optimum conditions derived from the multiple groups investigated in this study needs to be confirmed using primary human cells.

In conclusion, our study utilizes a 3D co-culture model of osteoblast-osteocyte connectivity in 3D to approximate the signalling observed within bone, while providing a system that is easier to apply.

## 4. Materials and Methods

### 4.1. Cell Culture

Three independent co-culture experiments were performed using both MG-63 osteoblastic and MLO-Y4 osteocyte-like cells. Prior to experiments the human osteoblast cell line MG-63 was cultured in monolayer until confluence. Cells from passage 16–20 were used for co-culture experiments at 70% confluence. The mouse osteocyte cell line MLO-Y4 was kindly provided by L.F. Bonewald [25]. Cells were cultured in monolayer and used for experiments between passage 39 and 44. The MLO-Y4 cell line was maintained as advised by the lab of L.F. Bonewald and phenotypic criteria of dendricity and high osteocalcin and E11 expression were utilized.

MG-63–MLO-Y4 co-cultures were prepared in type I collagen gels as previously described [18]. Briefly, 50 mg type I collagen was dissolved over night at 4 °C in 7 mM acetic acid to produce 2.5 mg type I collagen per ml solution. DMEM (10×, Gibco, Waltham, MA, USA) was mixed 1:1 with deionized water containing 22 g/L NaHCO_3_. This 5× DMEM was added 1:4 to the 2.5 mg/mL type I collagen on ice. The mixture was neutralized with 1 M Tris base (pH = 12.6) prior to the addition of cells. MLO-Y4 cells were seeded at a density of 1.5 × 10^6^ cells/mL into 250 μL gels. Each gel was prepared within one well of a 24-well plate. Gels were left to polymerize at 37 °C for 1 h. A layer of 1.6 × 10^5^ MG-63 cells was seeded on top of each gel. Co-cultures were performed cultured for 6 days in either (i) αMEM (Gibco) + 5% foetal calf serum (FCS; Biowest, Nuaillé, France) + Pen/Strep (10 kU Penicillin: 10 mg Streptomycin; Invitrogen, Waltham, MA, USA), or (ii) αMEM + insulin-transferrin-selenium-lipid supplementation (Gibco; serum free (SF) culture) + Pen/Strep—with or without the addition of 15 ng/mL TGFβ_3_ (Novartis Pharma AG, Basel, Switzerland). Each media group contained 3–4 replicates per time point (day 2, day 4 and day 6). Co-cultures were supplied with 1 mL media per gel. Media was exchanged every 48 h.

### 4.2. RT-qPCR

RNA samples were taken from co-cultures every 48 h, starting on day 2. Cell lysis of osteoblasts and osteocytes was performed separately using TRIzol as previously described (Molecular Research Centre, Cincinnati, OH, USA) [18]. Briefly, gels were incubated first for 10 s with TRIzol to collect the surface osteoblasts, prior to a separate TRIzol incubation to collect the embedded osteocytes. Osteoblast and osteocyte RNA samples were isolated according to manufactures instructions. Reverse transcription (RT) was performed with 0.5 μg RNA per reaction using random hexamers and Superscript III (all Invitrogen) according to manufacturer’s protocol. Quantitative polymerase chain reaction (qPCR) was performed on a Stratagene MX3000 using incorporation of Sybr® green (Sigma-Aldrich, St. Louis, MO, USA). Glyceraldehyde 3-phosphate dehydrogenase (GAPDH) was chosen as housekeeping gene for the human cell line MG-63, as its expression was the least affected by the treatments. 18S ribosomal RNA (18SrRNA) was chosen as the MLO-Y4 housekeeping gene for the same reason. Expression levels of osteoblastic and osteocytic marker genes (Table 1) were made relative to levels at day 2 samples in αMEM + FCS after normalization to housekeeping genes.

### 4.3. Immunocytochemical Staining

The presence of β-actin and the C-terminal propeptide of type I collagen (ProCI; M38 DSHB) was detected via immunocytochemistry. Co-cultures were fixed in 1% paraformaldehyde (Sigma-Aldrich, St. Louis, MO, USA) in 0.05 M phosphate buffered saline for 30 min at 4 °C on culture days 2, 4 or 6. Cryosections (thickness of 10 μm) from the central region of the co-cultures were prepared. Fluorescence labelling of β-actin (phalloidin, Alexa 488), together with ProCI (Alexa 594; all Invitrogen) and DAPI was performed.

### 4.4. Viability and Cell Death

Cell viability of osteoblasts and osteocytes within co-cultures was qualitatively determined using the lactate dehydrogenase assay (Sigma-Aldrich). Therefore, unfixed cryo-sections (12 μm) from co-cultures on culture day four were prepared. The LDH assay on unfixed cryo-sections was performed as previously described [30].

Cell death was quantified using the Terminal deoxy nucleotidyl transferase dUTP-nick-end-labelling (TUNEL) assay. Seven images per media group and per time point were taken from the centre region of the collagen gels. Dead osteocytes and residual (alive) osteocytes were manually counted and percentages calculated. Controls were performed according to manufactures instructions (DeadEnd™ Fluorometric TUNEL System was from Promega, Fitchburg, MA, USA). The positive control was pre-digested with DNase I prior to TUNEL labelling. The negative control was performed without the addition of the Terminal Deoxynucleotidyl Transferase enzyme.

### 4.5. Statistical Analysis

Statistical analyses were carried out using SPSS 16.0 software package (IBM, New York, NY, USA). The analysis of the RT-qPCR data was performed on the dCT datasets. Normal distribution was confirmed by Kolmogorov–Smirnov test. One-way ANOVA and Tukey post-hoc tests were used to compare the data sets. Primary outliers and extremes were determined as being numerically distant from the rest of the data (outliers > 1.5 times the interquartile range, extremes > 3 time the interquartile range) and were removed from the analysis.

The TUNEL assay data was not normally distributed. Therefore, Mann-Whitney Rank Sum Test and Bonferroni post-hoc test were used as statistical analyses. A *p*-value of below 0.05 was considered statistically significant.

## Figures and Tables

**Table 1:** RT-qPCR was performed to investigate relative expression levels of human GAPDH, human Runx2 and human osteocalcin in MG-63, as well as mouse 18SrRNA, mouse E11 and mouse osteocalcin in MLO-Y4. The table shows the primer sequences used.

Gene	Forward Primer	Reverse Primer
human GAPDH	GGT ATC GTG GAA GGA CTC ATG A	GGC CAT CCA CAG TCT TCT G
human Runx2	GTG GAC GAG GCA AGA GTT TC	TTC CCG AGG TCC ATC TAC TG
human osteocalcin	GGC AGC GAG GTA GTG AAG AG	GAT CCG GGT AGG GGA CTG
human type I collagen	CCC TGG AAA GAA TGG AGA TGA T	ACT GAA ACC TCT GTG TCC CTT CA
mouse 18SrRNA	GCA ATT ATT CCC CAT GAA CG	GGC CTC ACT AAA CCA TCC AA
mouse E11	AAG ATG GCT TGC CAG TAG TCA	GGC GAG AAC CTT CCA GAA AT
mouse osteocalcin	CAGACAAGTCCCACACAGCA	GAA AT

## Abbreviations

2/3D	Two-/Three-dimensional
TGFβ_3_	Transforming growth factor beta 3
RT-PCR	Reverse transcribed polymerase chain reaction
ECM	Extracellular matrix
SF	Serum free
FCS	Fetal calf serum
